# Pyroelectric synthesis of Au/Pt bimetallic nanoparticles–BaTiO_3_ hybrid nanomaterials†

**DOI:** 10.1039/d0ra00648c

**Published:** 2020-06-12

**Authors:** Liren Wang, Han Wang, Yanming Liu, Xinyu Wang, Peng Tao, Wen Shang, Benwei Fu, Chengyi Song, Tao Deng

**Affiliations:** State Key Laboratory of Metal Matrix Composites, School of Materials Science and Engineering, Shanghai Jiao Tong University 800 Dong Chuan Road Shanghai 200240 P.R.China chengyi2013@sjtu.edu.cn dengtao@sjtu.edu.cn

## Abstract

This paper introduces an approach to synthesize bimetallic nanoparticles under an alternating temperature field in aqueous solution. During the synthesis, pyro-catalytic barium titanate is used as the substrate to reduce the metallic ions dispersed in the solution due to the generated charges at the surface of pyro-materials under temperature oscillation. Chloroauric acid and potassium tetrachloroplatinate are used as precursors to produce gold/platinum bimetallic nanoparticles through a pyro-catalytic process. Transmission electron microscopy characterization, in combination with energy dispersive X-ray spectroscopy mapping, demonstrates that the bimetallic nanoparticle is composed of an Au core and Au/Pt alloy shell structure. Compared to the conventional approaches, the pyroelectric synthesis approach demonstrated in this work requires no toxic reducing agents and waste heat can be used as a thermal energy source in the synthesis. Hence, it offers a potential “green” synthetic method for bimetallic nanoparticles.

## Abstract 2

A “green” synthetic approach to Au/Pt bimetallic nanoparticles under an alternating temperature field.

## Graphical abstract

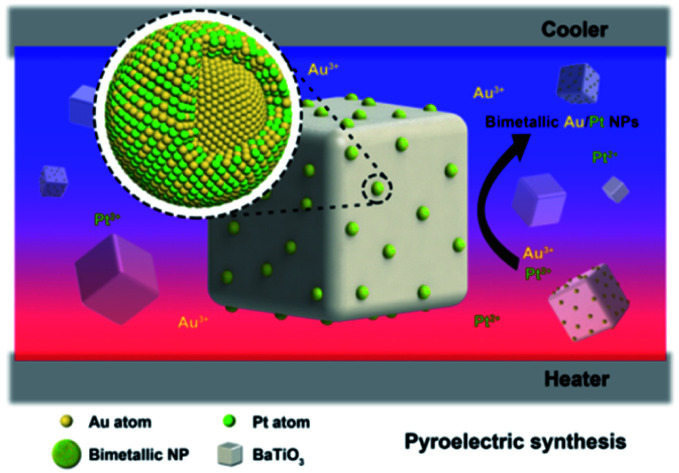

## Introduction

Bimetallic nanoparticles (NPs) have received wide attraction due to their electrical and optical properties, and their applications in catalysis.^1–8^ Over the past decades, the synthesis of bimetallic NPs with different structures has gained much attention, and significant efforts have been made in controlling the composition, size and morphology *via* various synthetic processes.^9,10^ Conventional methods for the preparation of bimetallic NPs can be divided into two categories: physical^11^ and chemical techniques.^12–14^ The typical colloidal route is the most widely adopted strategy,^12–14^ which requires the reduction of metal ions into neutral atoms followed by the deposition of metallic atoms onto the growing particles. Au/Pt NPs are one of the most important types of bimetallic NPs because of their special catalytic properties. The synthesis of bimetallic Au/Pt NPs, however, is a significant challenge as Au is hard to be miscible with Pt.^15^ In addition, the traditional synthesis approach involves the use of some organic reductants and stabilizers, which are usually toxic and expensive.^16^

Recently, there have been growing researches on applying pyro-catalytic materials to drive chemical redox reactions utilizing the pyroelectric effect. Pyroelectric effect is the property of the materials with polar point symmetry that shows a spontaneous polarization without the existence of an applied electric field^17^. Once the ambient temperature of the material fluctuates, there will be a variation of the direction of spontaneous polarization, which further leads to the separation of surface-bounded compensation charges. As a result, excess compensation charges generated on the surface of pyro-catalytic materials. These generated charges may later be applied in the subsequent chemical reaction. Various reports have shown the efforts in carrying out reduction reactions through pyroelectric effect. Jiang *et al.* reported the use of pyro-catalytic BiFeO_3_ NPs to generate reactive oxygen species through temperature alternation for the degradation of the organic waste in wastewater.^18^ Recently, Huilin *et al.* used pyro-catalytic 2D few-layer black phosphorene for hydrogen evolution under thermal cycling between 15 °C and 65 °C. Compensation positive and negative charges were generated at the surface of the pyro-catalytic material and further transferred to the hydrogen ion to participate in the redox reaction when the system was subjected to the ambient cold–hot alternation.^19^ Min *et al.* also adopted a porous BaTiO_3_ NP composite membrane to degrade dye molecules under periodic illumination of solar light. Localized heat was generated on the membrane due to the high efficient light-to-heat conversion process when light was on. On the other hand, the porous structure resulted in rapid interfacial evaporation when light was off, which cools down the heated membrane. Such rapid temperature oscillation could reach an instantaneous rate of temperature change of 13 °C s^−1^ during the degradation process, showing a 4 times higher efficiency of pyro-catalysis of the composite membrane compared to the dispersed BaTiO_3_ solution.^20^ These reports reveal that pyroelectric nanomaterials can provide reducing agents to reduce species that are usually in oxidation states. In our previous work, we have demonstrated that barium titanate (BaTiO_3_, BTO) NPs could be served as pyroelectric support to synthesize different metallic NPs (*e.g.* Au, Pt, Pd NPs) in aqueous solution or organic solvent.^21^ With the participation of compensation charges generated at the surface of pyroelectric materials, the metallic ions in the solution could be reduced into corresponding metal elements and then form metallic NPs. The advantages of this method are apparent that toxic reducing agents are not required as in the conventional approaches, and waste heat can be utilized as an energy source to drive these redox reactions.

On the other hand, comparing to single-component metal oxide particles, the hybrid structure with the deposition of noble metal NPs shows a better performance in various applications such as optics^22^ and photocatalysis^23^ due to the stronger absorption in the range of visible light and the slower recombination rate of photogenerated electron–hole pairs.^24^ Furthermore, the Au/Pt bimetallic NPs show a superior performance rather than Au monometallic NPs due to the electronic transfer from Au to Pt. For example, Gallo *et al.*^25^ reported the bimetallic Au0.5–Pt0.5/TiO_2_ photocatalyst shows the highest hydrogen production rate among Au–TiO_2_ and Pt–TiO_2_ under UV light and simulated sunlight irradiation. In the field of organic synthesis, Shen *et al.*^26^ found that using Au/Pt–TiO_2_ as the catalyst to oxidize glycerol into lactic acid gave higher yield and product selectivity than the monometallic Au/TiO_2_, Pt/TiO_2_, and the physical mixture of Au/TiO_2_ with Pt/TiO_2_. These advantages imply that he Au/Pt–BTO has a great potential in the application of catalysis.

**Fig. 1:**
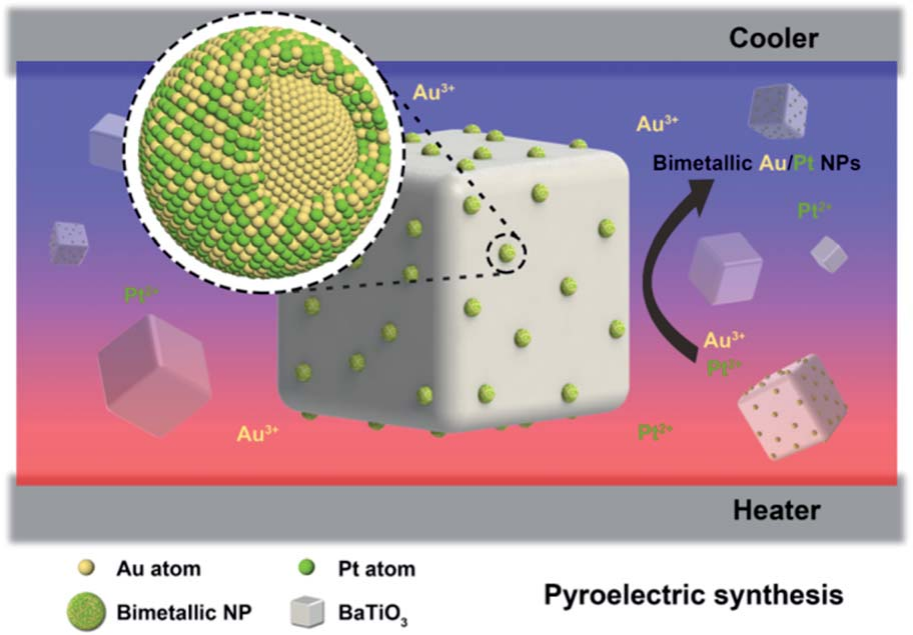
The schematic illustration of the structure and composition distribution of bimetallic Au/Pt NPs, which grew on the pyroelectric BTO NPs under temperature fluctuation.

In this study, we explored the synthesis of BTO–Au/Pt hybrid nanomaterials in aqueous solution under temperature alternation (Fig. 1). BTO particles were modified by (3-mercaptopropyl)trimethoxysilane (MTS) first to enhance the reaction activity. The synthesis employed chloroauric acid (HAuCl_4_) together with potassium tetrachloroplatinate (K_2_PtCl_4_) as precursors, and potassium hydroxide was added together to adjust the pH value. Characterization methods, which include transmission electron microscopy (TEM) and energy dispersive X-ray spectroscopy (EDS) mapping, have been applied to determine the structure of the product. Results of the characterization showed that the bimetallic Au/Pt NPs with an average particle size of 10 nm are formed on the surface of BTO and had an Au core and Au/Pt alloy shell structure. We expect that this green chemically synthetic procedure can pave an alternative way to synthesize bimetallic NPs, metallic compositions of which are not easily miscible.

## Materials and methods

### Materials

Potassium tetrachloroplatinate (K_2_PtCl_4_, 98%), potassium hydroxide (KOH) and (3-mercaptopropyl)trimethoxysilane (C_6_H_16_O_3_SSi, MTS, 95%) were purchased from Aladdin Company. Ethanol (CH_3_CH_2_OH, 99.7%) and chloroauric acid (HAuCl_4_, 47.8%) were obtained from Sinopharm Chemical Reagent Company. The BTO particles were pre-synthesized *via* a hydrothermal method.^27^ All the chemicals used were of analytical grades without further purification.

### Synthesis of Au/Pt bimetallic NPs

Conventional chemical synthesis methods for the preparation of Au/Pt NPs use metallic salts HAuCl_4_ and K_2_PtCl_4_ as the reactant, together with aqueous NaBH_4_ or formaldehyde as reducing agent. To direct the reaction and crystal growth, capping agents were often added into the reaction.

**Fig. 2:**
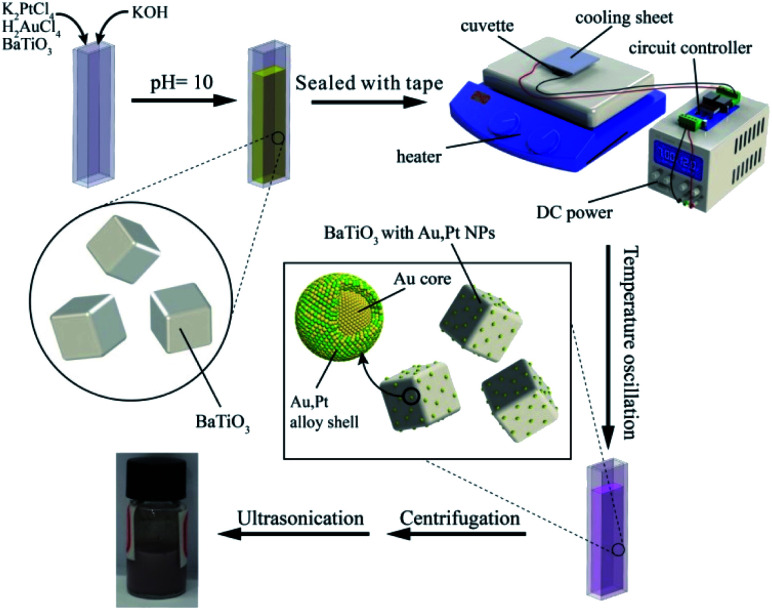
Schematic illustration of the experimental setup of pyroelectric synthetic procedure and the pyro-catalytic reduction process of Au/Pt bimetallic NPs on the surface of the BTO particle.

In this experiment, we substituted the pyro-catalytic BTO particles for the traditional reductant and kept the other conditions the same. Fig. 2 gives a schematic illustration of the pyroelectric synthesis process of Au/Pt bimetallic NPs. The glass cuvette filled with reaction solution was placed on the hot plate. One side was glued to the cooling sheet by silver thermal grease, while the other side was in close contact with the heating plate. The cooling sheet was connected to a DC power supply with a voltage of 12 V. Time-dependent temperature oscillation was achieved by controlling the heater to constantly heat the glass cell, while the cooler was switched on/off every 15 seconds through the use of a circuit controller. Fig. S1† showed that the average temperature of the solution was around 91 °C with average peak variation rate of 0.75 °C s^−1^ during the running period.

The pre-synthesized pyro-catalytic BTO particles were modified by MTS to enhance the surface reaction activity. 10 mg of synthesized BTO particles were dissolved in 10 mL ethanol, followed by adding 100 μL MTS into the mixture dropwise. The mixture was ultrasonicated for 20 minutes, subsequently centrifuged out at 8000 rpm for 5 minutes and washed with ethanol several times. After being heated in the furnace at 60 °C for 12 hours, the dried product was dispersed in 10 mL of deionized water for stock.

Au/Pt bimetallic NPs were generated by a reduction reaction using HAuCl_4_ and K_2_PtCl_4_ as precursors in a PTFE sealed cuvette. In a typical synthesis, 800 μL of as-prepared BTO emulsion was first added into the glass cuvette. Subsequently, 380 μL of HAuCl_4_ solution and 380 μL of K_2_PtCl_4_ solution were mixed together in the glass cuvette. The concentrations of the metallic salts were both 20 mmol L^−1^ to ensure the equivalent molar concentration of Au and Pt. After the addition of 40 μL 1 M solution of KOH to adjust the pH to 10, the cuvette was sealed with PTFE tape and placed in the experimental set-up. The reaction solution was mixed homogeneously and heated to maintain the reaction temperature between 88 °C and 95 °C, with average peak variation rate of 0.75 °C s^−1^ for 2 hours. Under the alternating temperature field, the colour of the reaction solution turns from yellow to pink, implying that the metallic salts had been reduced. The solids were separated from the mixture by centrifugation at 12 000 rpm for 5 minutes and washed with deionized water twice after the reaction.

Pure Au NPs or pure Pt NPs on BTO were generated by our published method.^21^

### Methods

To demonstrate the successful synthesis of Au/Pt bimetallic NPs anchored on the surface of BTO particles, the as-prepared NPs were re-dispersed in deionized water and further analysed using TEM (TALOS F200X, JEOL, Japan), which was operated at an accelerating voltage of 200 kV and equipped with EDS. In the TEM characterization, 10 μL of the product solution was drop-casted onto the carbon-coated copper grids followed by solvent evaporation in the air at room temperature before the characterization. The EDS was employed for elemental composition and distribution of the synthesized product. The distribution of the elements within a single particle was determined by identification of the elemental peaks observed in the EDS spectrum at each pixel during mapping.

## Results and discussion

### Structural characterization of Au/Pt bimetallic NPs

**Fig. 3:**
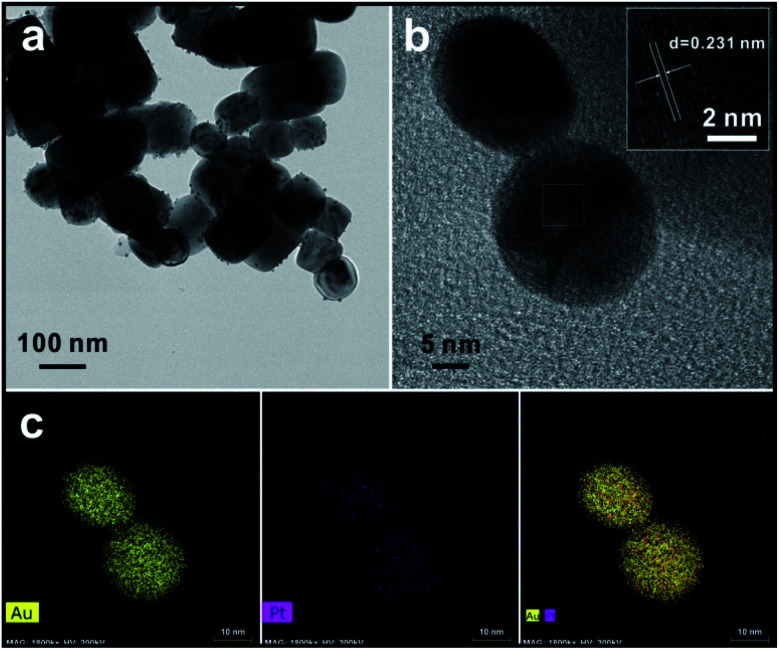
(a and b) TEM images of Au/Pt bimetallic NPs on the surface of BTO with different magnification, and the image inset in (b) is the HRTEM image of Au/Pt bimetallic NPs with measured lattice distance. (c) EDS mapping images of Au/Pt bimetallic NPs on the surface of BTO. Colored pixels indicate where the element was detected above background.

Fig. 3 shows TEM images of the as-synthesized product after the 2 hour reaction. As shown in Fig. 3a, the cubic BTO particle substrates have a size of about 100 nm. After the reduction reaction takes place on the surface of the BTO particles, the spherical shaped Au/Pt bimetallic NPs are uniformly distributed on the surface of BTO and have relatively narrow size distribution with a size of ∼10 nm (Fig. S2†). This relatively narrow size distribution indicates that the formation of bimetallic NPs undergoes a two-step process: heterogeneous nucleation of metallic seeds followed by the growth of the nuclei as the metallic ions are continuously reduced during the reaction. The crystal seeds proceed to grow and finally generate the relatively monodispersed particles.

A high-resolution transmission electron microscope (HRTEM) was also applied to measure the lattice spacing of the bimetallic NPs. For standard pure Au and standard pure Pt, the interplanar distances of (111) facet are 0.235 nm and 0.226 nm, respectively (Fig. S3†). From the inset HRTEM image shown in Fig. 3b and S4,† a lattice spacing of 0.231 nm depicted by parallel lines and arrows was discovered. The value between 0.235 nm and 0.226 nm indicates the formation of the Au/Pt alloy with an FCC structure, through which the lattice parameter can be calculated as 4.001 Å. Based on the above evidence, the bimetallic NP might be an alloy structure (Fig. S5†). Lou *et al.* showed the dependence of the lattice parameter for Au/Pt alloy on the relative composition percentage of Au, and they reported that the Au/Pt alloy with a lattice parameter *a* = 4.001 Å had a composition of 50% Au,^28^ which means that the Au/Pt bimetallic NPs synthesized in this work should have also the atomic ratio of ∼1 : 1. The ICP-OES characterization was also conducted to determine the composition of the bimetallic NPs, which also implied the ratio of Au : Pt is 3 : 1. To better study the metal segregation of the synthesized product, EDS mapping was used to analyse the overall composition and element distribution of a single particle chosen arbitrarily. The crystal lattice could be directly seen throughout the entire particle. Results showed in Fig. 3c suggest that Au and Pt compositions are homogeneously dispersed on the surface of bimetallic NPs. The atomic ratio of Au : Pt obtained from the EDS mapping result, however, is 3 : 1. It implies the bimetallic NP should be an Au-core and Au/Pt alloy shell structure. Such unique structure may due to the reactivity difference between Au and Pt. The hypothesized synthetic mechanism will be discussed in the following section.

### Pyroelectric synthesis mechanism of alloy NPs

**Fig. 4:**
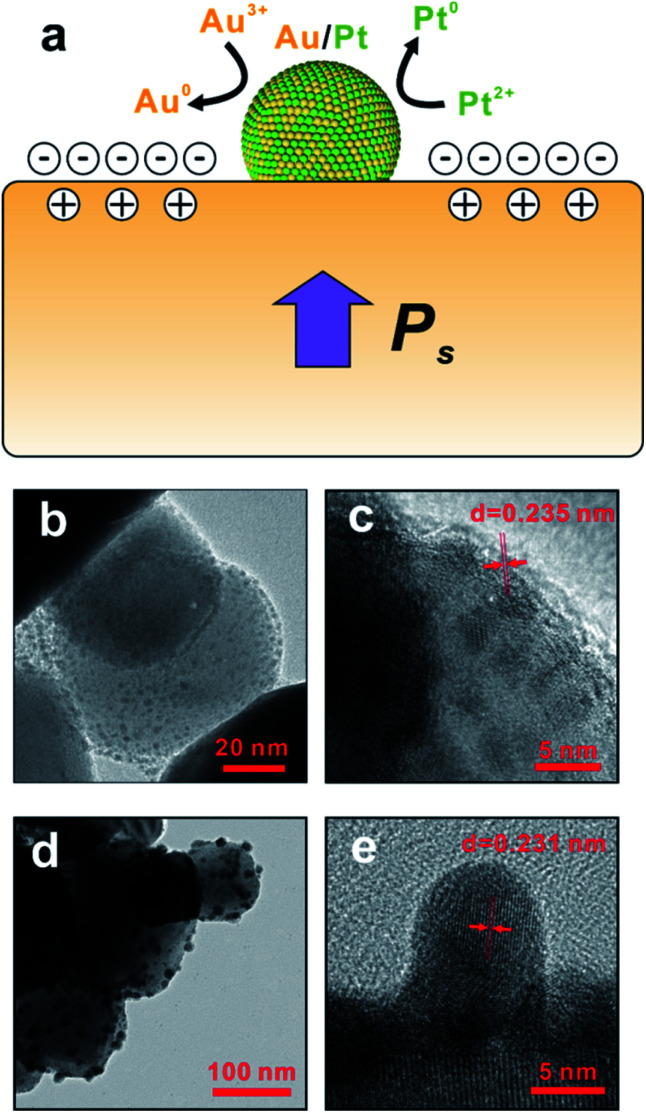
(a) Scheme illustration of the pyro-catalytic reaction mechanism, *i.e.*, the cold–hot alternation excitation of a pyroelectric material and the subsequent redox reaction on the surface. (b) low-magnified TEM image of the growth of the particles at early stage; (c) early-stage HRTEM image of the particle with lattice spacing. (d) low-magnified TEM image of the growth of the Au/Pt NPs at late stage; (e) late-stage HRTEM image of the Au/Pt NPs with lattice spacing.

Compared to the conventional synthetic methods, the pyroelectric approach explored in this paper utilizes pyro-catalytic NPs instead of other reducing agents that are usually toxic and expensive. In this paper, when the ambient temperature is constant (Δ*T* = 0), the level of spontaneous polarization of BTO remains at a constant value, and thus the total amount of compensation charges attracted onto the surface of BTO also remains steady, which completely screens the polarization charges of BTO NPs. When BTO is heated at a temperature alternation rate of 0.75 °C s^−1^ (Δ*T* > 0), the internal dipoles within the BTO lose their orientation, which lowers the level of the spontaneous polarization and leaves excess compensation charges *q*^+^ and *q*^−^ on the BTO surface.^29^Fig. 4a shows a schematic diagram of the working principle of the pyroelectric redox reaction. The temperature oscillation triggers the pyroelectric effect. As the ambient temperature changes, a net change of the electric dipole moment of the pyro-catalytic materials occurs, which causes the excess charge compensation on the surface of the pyro-catalytic materials. In order to maintain the electro-neutrality, redox reactions subsequently occur to consume the extra charges. As the reduction of the Au^3+^ ions of AuCl_4_^−^ takes place only in alkaline environment,^30^ KOH was thus added to adjust the pH value of the solution. During the temperature fluctuation, the redox reactions happened in both the heating and cooling processes of the thermal cycle so these reactions proceed continuously as long as there is temperature change of the solution. According to our previous work about the synthesis of metal–BTO hybrid NPs, the surface-absorbed metallic ions around the BTO tends to react with the excess compensation charge *q*^−^ to balance extra electric charges, resulting in the generation of corresponding Au/Pt bimetallic NPs on the surface of BTO as the reaction product.^21^ The reaction can be expressed as the following eqn (1) and (2).

1 [AuCl_4_]^−^ + 3*q*^−^ → Au + 4Cl^−^ *E*^*θ*^ = +1.002 V

2 [PtCl_4_]^2−^ + 2*q*^−^ → Pt + 4Cl^−^ *E*^*θ*^ = +0.755 V

With a higher electrode potential, the Au salt is more likely to be reduced by the compensation charge *q*^−^ rather than the Pt salt. It is believed that the generated Au atom forms the Au NP-core of the bimetallic NPs first.^31,32^ With the reaction proceeds, MTS plays an important role in the co-reduction of both of Pt atoms and Au atoms from their salts to form Au/Pt shell structure. The use of MTS for the modification of the surface of BTO was to enhance the productivity of NPs. The –SH groups can form chelates with the Au, Pt metal ions through Au–S and Pt–S bonds^33^ in order to chemisorb the metallic ions during the synthesis. The metallic ions will be attracted to the surface of BTO and more likely to react with the excess compensation charges. MTS is also applied as the capping agent, which helps to minimize NP aggregation in the solution^34^ and stabilizes the synthesized bimetallic NP seeds that were generated in the early stage of the reduction reaction and subsequently grew up as NPs.

Furthermore, to investigate the impacts of MTS during the synthesis, Au/Pt hybrid NPs were synthesized by using the bare BTO substrate without the modification of MTS. Fig. S6† showed the TEM image and size distribution of the NP. The mean diameter was about 1.7 nm, which was much smaller than the particles synthesized with the addition of MTS. The relatively small sizes of NPs might be ascribed to the lower growth rate of nuclei in the reaction solution. Hence, MTS can serve as good nucleating agent for metallic particle seeds, and it has impact on the reduction rate of nanoparticles on the surface of BTO.

The time-dependent experiment (Fig. 4b–e) shows the evolution of bimetallic NP growth during the reaction, and also provides compelling evidence of the formation mechanism of bimetallic NPs. The relatively small particle in Fig. 4b has a size of 3 nm and lattice spacing of 0.235 nm (Fig. 4c), indicating the formation of Au core during the early state of synthesis of bimetallic NPs. At the late stage of the synthesis, the particle size grows to 8 nm as shown in Fig. 4d. The lattice spacing reduced to 0.231 nm (Fig. 4e), which suggests the Au/Pt alloy shell is formed.

It is notable that the size of the NP changes significantly during different reaction period. To investigate the impact of reaction time on the sizes of resulting NPs, controlling experiments with different reaction time of 30 minutes and 4 hours were conducted. The average diameter of the product particles after reacting for 30 min was about 1.7 nm (Fig. S7b†), whereas the mean size of Au/Pt bimetallic NPs after 4 hour-reaction was about 10.6 nm (Fig. S7d†). When the reaction time was 30 minutes, bimetallic seeds were still in the early state of the synthesis, the insufficient reaction time leads to the relatively small particle size. MTS can also serve as good capping reagent for the nanoparticles. They can stabilize nanoparticles and avoid the merge of neighbouring nanoparticles.

The positive compensation charge *q*^+^ in the solution undergoes an oxidation process and reacts with the surface-absorbed OH^−^ ions to produce ·OH according to the following reaction^35^ (eqn (3)).

3 OH^−^ + *q*^+^ → ·OH

Afterward, when the temperature approaches to stabilization (Δ*T* = 0), the reaction stops and the system returns to the initial state. Similarly, when BTO is cooled at the rate of 0.75 °C s^−1^ (Δ*T* < 0), its spontaneous polarization level increases. The shortage of compensation charges drives the BTO to generate electric charges and repeats the redox-reaction process described by eqn (1)–(3). A controlled experiment was carried out to further verify the importance of temperature fluctuation during the pyroelectric synthesis. The cooler in the experimental setup was removed in order to conduct the reaction at a constant temperature without temperature oscillation. Fig. S8† showed that no obvious production of metallic particles was observed after the 2 hour reaction.

## Conclusions

In conclusion, we demonstrate an alternative approach to synthesize Au/Pt bimetallic NPs using pyro-catalytic BTO NPs. With the temperature alternation, the pyroelectric effect induces a spontaneous change of the polarization intensity and leads to the excess compensation charges on the surface of BTO, which subsequently react with the metallic ions to generate bimetallic NPs. The characterization results show the bimetallic NPs have an alloy structure on the surface with an average size of 10 nm. The results offer an alternative pathway for the preparation of Au/Pt bimetallic NPs and can be extended to other redox reactions utilizing temperature fluctuation.

## Conflicts of interest

There are no conflicts to declare.

## Supplementary Material

RA-010-D0RA00648C-s001
