# Supplementary material for: Pyroelectric synthesis of Au/Pt bimetallic nanoparticles–BaTiO3 hybrid nanomaterials
Source: RSC Adv. 2020 Jun 12;10(38):22616–21. doi: 10.1039/d0ra00648c (PMC9054579; doi:10.1039/d0ra00648c)
Supplement: RA-010-D0RA00648C-s001 [file RA-010-D0RA00648C-s001.pdf]

Electronic Supplementary Material (ESI) for RSC Advances.
This journal is © The Royal Society of Chemistry 2020

Supporting information for:

Pyroelectric Synthesis of Au/Pt Bimetallic Nanoparticles-$BaTiO_3$ Hybrid Nanomaterials

Liren Wang, Han Wang, Yanming Liu, Xinyu Wang, Peng Tao, Wen Shang, Benwei Fu, Chengyi Song*and Tao Deng*

State Key Laboratory of Metal Matrix Composites, School of Materials Science and Engineering, Shanghai Jiao Tong University, 800 Dong Chuan Road, Shanghai 200240, P.R.China.

Corresponding author: chengyi2013@sjtu.edu.cn; dengtao@sjtu.edu.cn

1. Supplementary Data (Figures S1-S8)

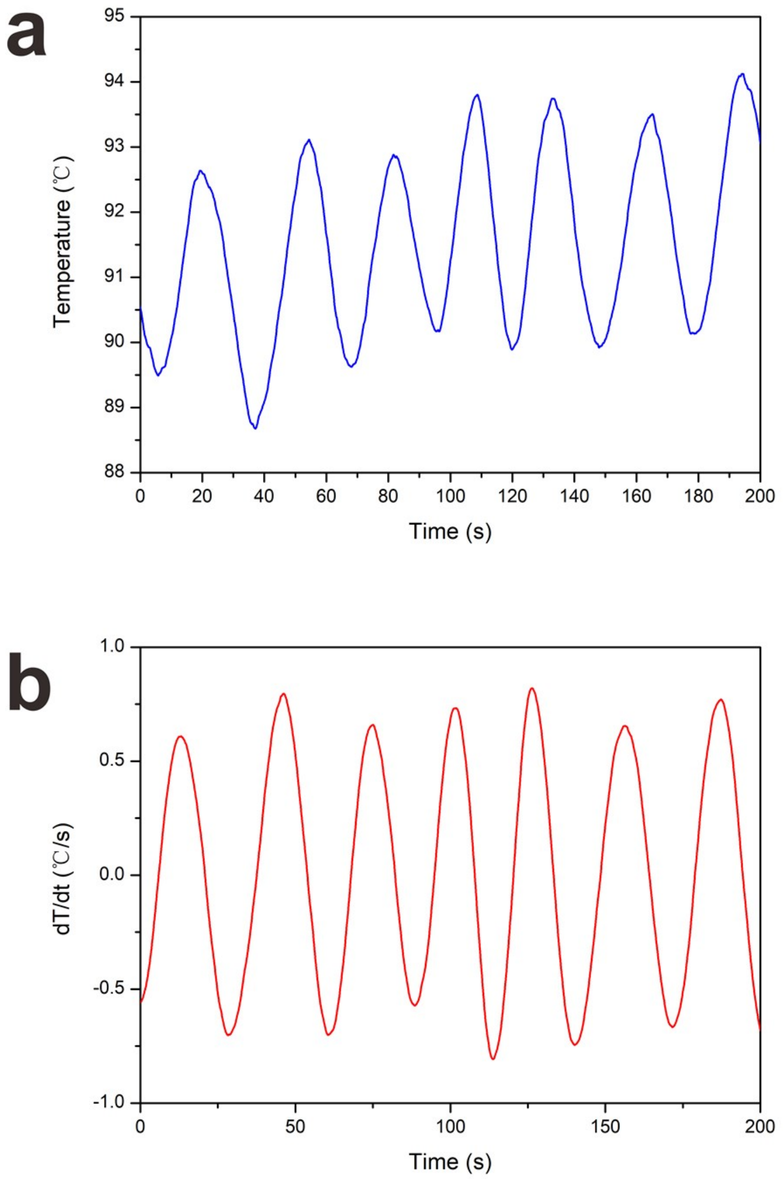

Figure S1. a) Temperature curve of the solution recorded by the thermal couple and b) the variation rate of temperature oscillation during the synthetic process.

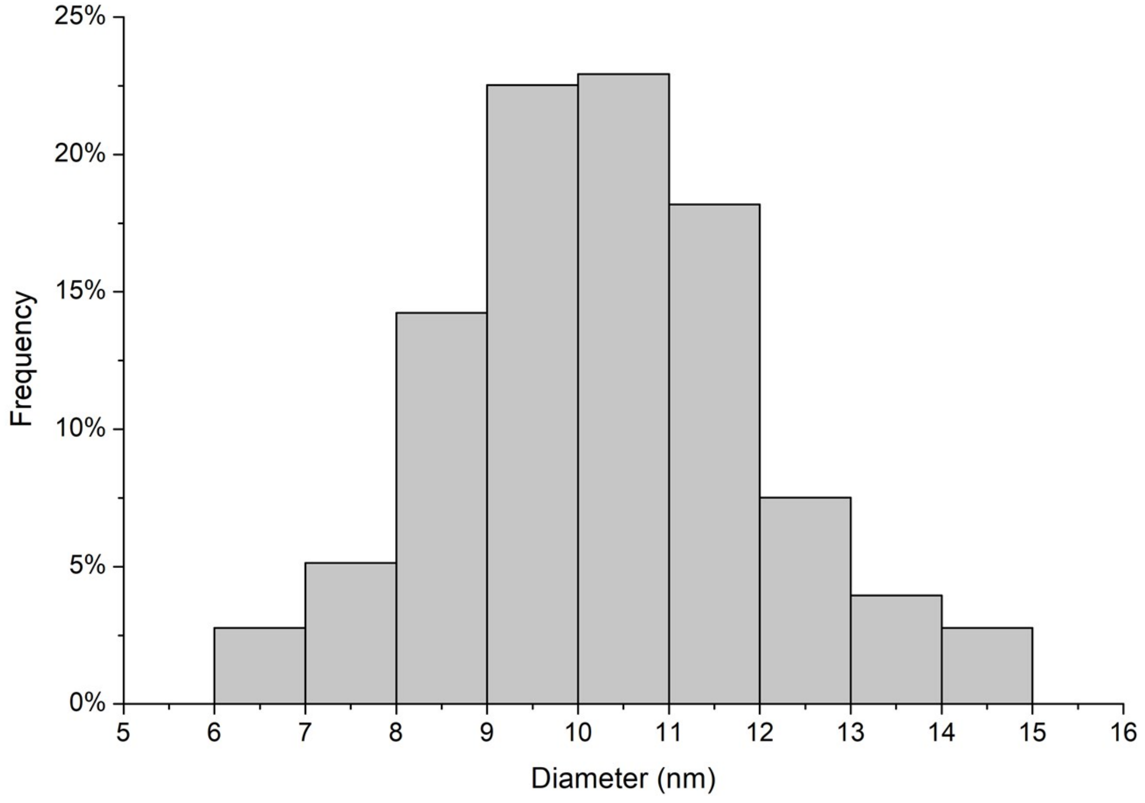

Figure S2. Size distribution of Au/Pt bimetallic NPs (based on 256 counts; NP diameter = 10.26 ± 1.69 nm).

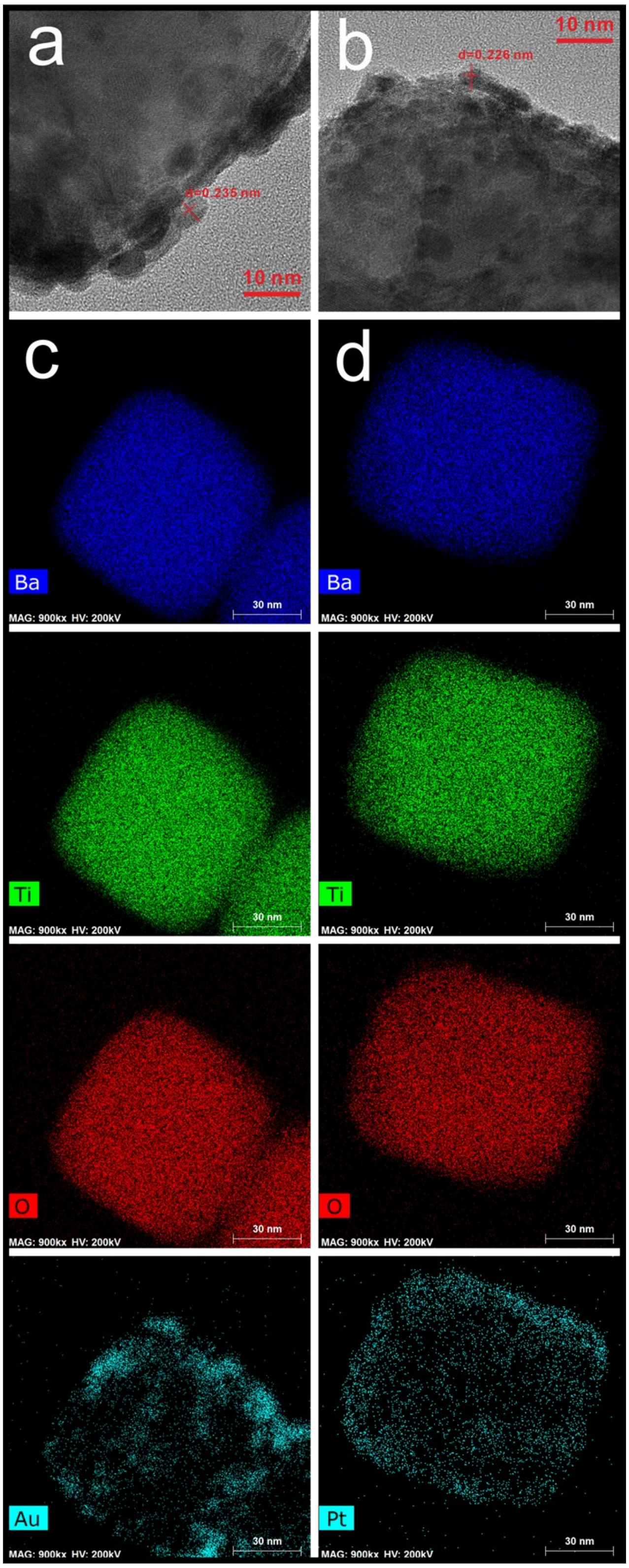

Figure S3. a) and b) HRTEM images of pure Au and Pt metallic nanoparticles on the surface of BTO with measured lattice distance, respectively. c) and d) EDS mapping images of pure Au and Pt metallic nanoparticles on the surface of BTO, respectively. Colored pixels indicate where the element was detected.

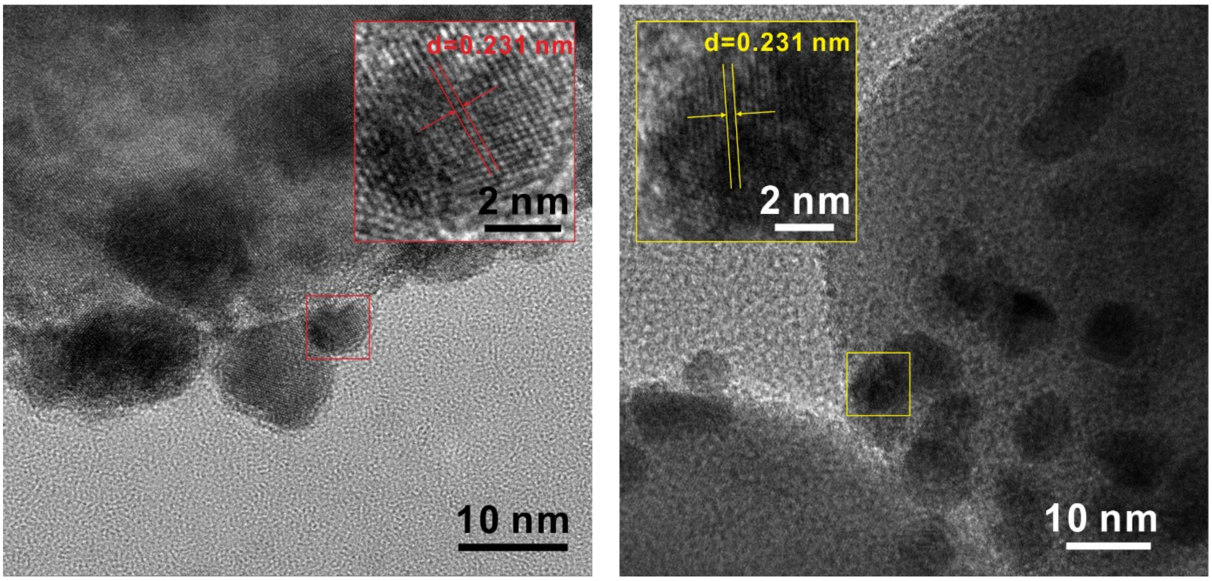

Figure S4. A lattice spacing of 0.231 nm depicted by parallel lines and arrows was measured on different Au/Pt bimetallic nanoparticles.

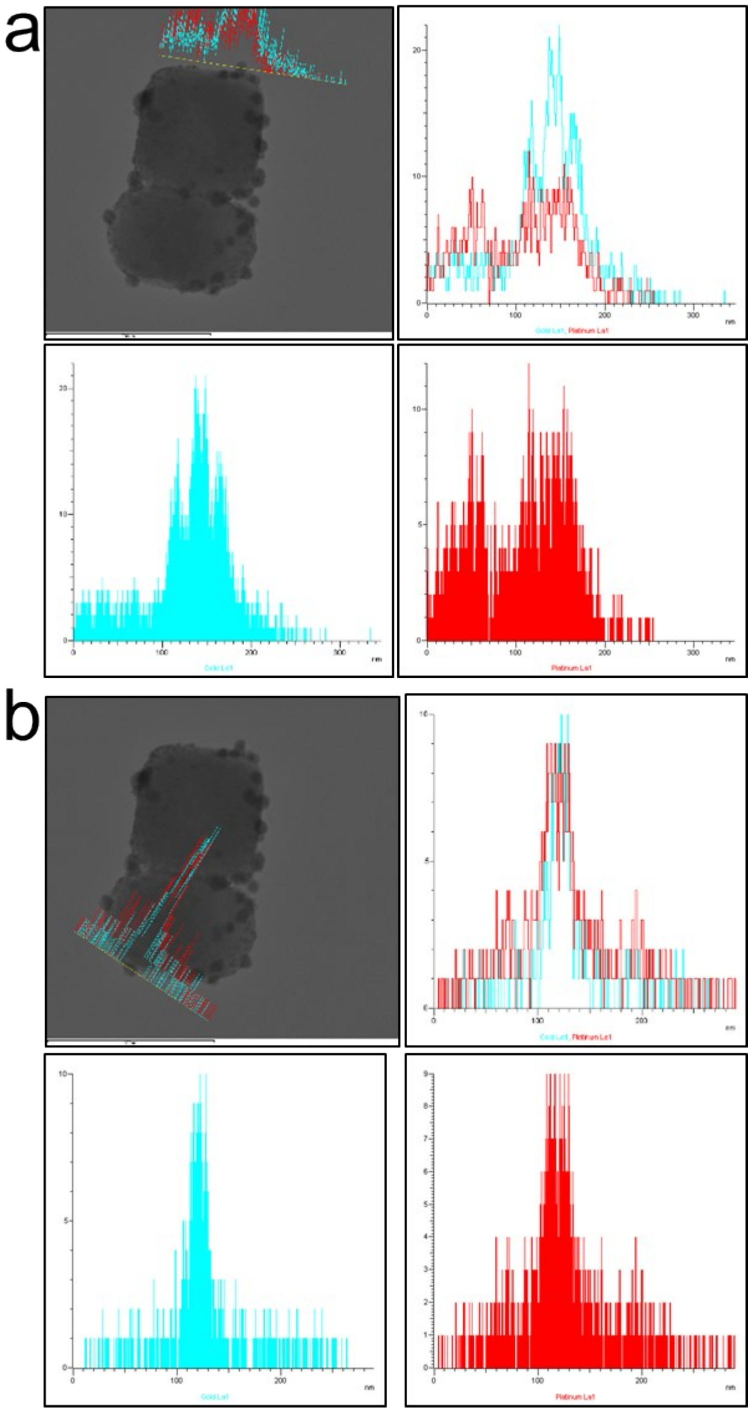

Figure S5. a) and b) EDS linear scan results for the elements of Au/Pt bimetallic nanoparticles. The intensity of the peaks represents the content of different elements; overlapped part of the peaks that is corresponding to the composition of the nanoparticle indicates both Au and Pt elements are detected, which proved that the surface of a bimetallic nanoparticle was an alloy of Au/Pt.

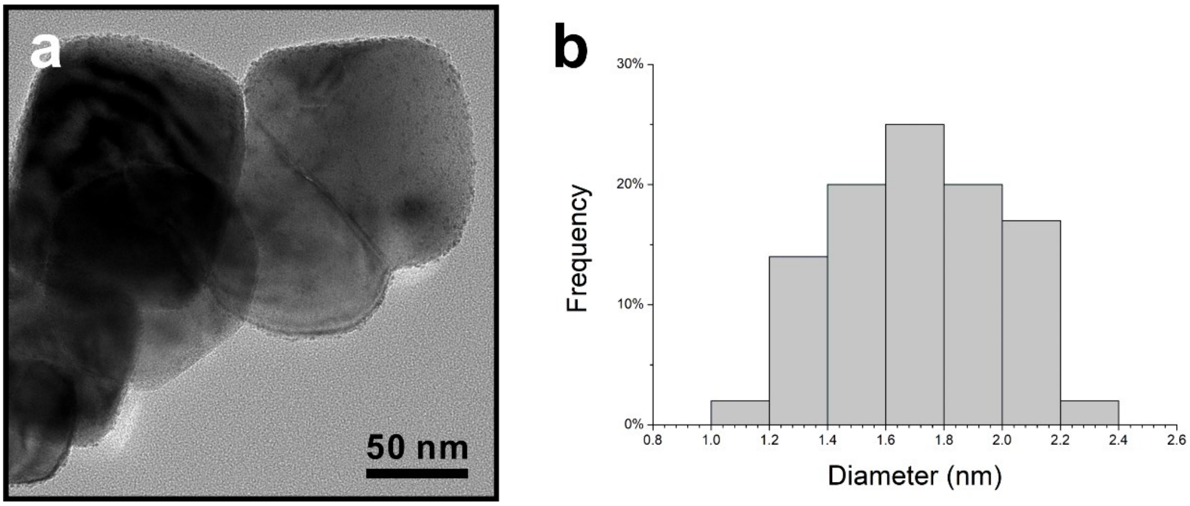

Figure S6. a) TEM image and b) size distribution of Au-BTO hybrid NPs synthesized by BTO substrate without the modification of MTS.

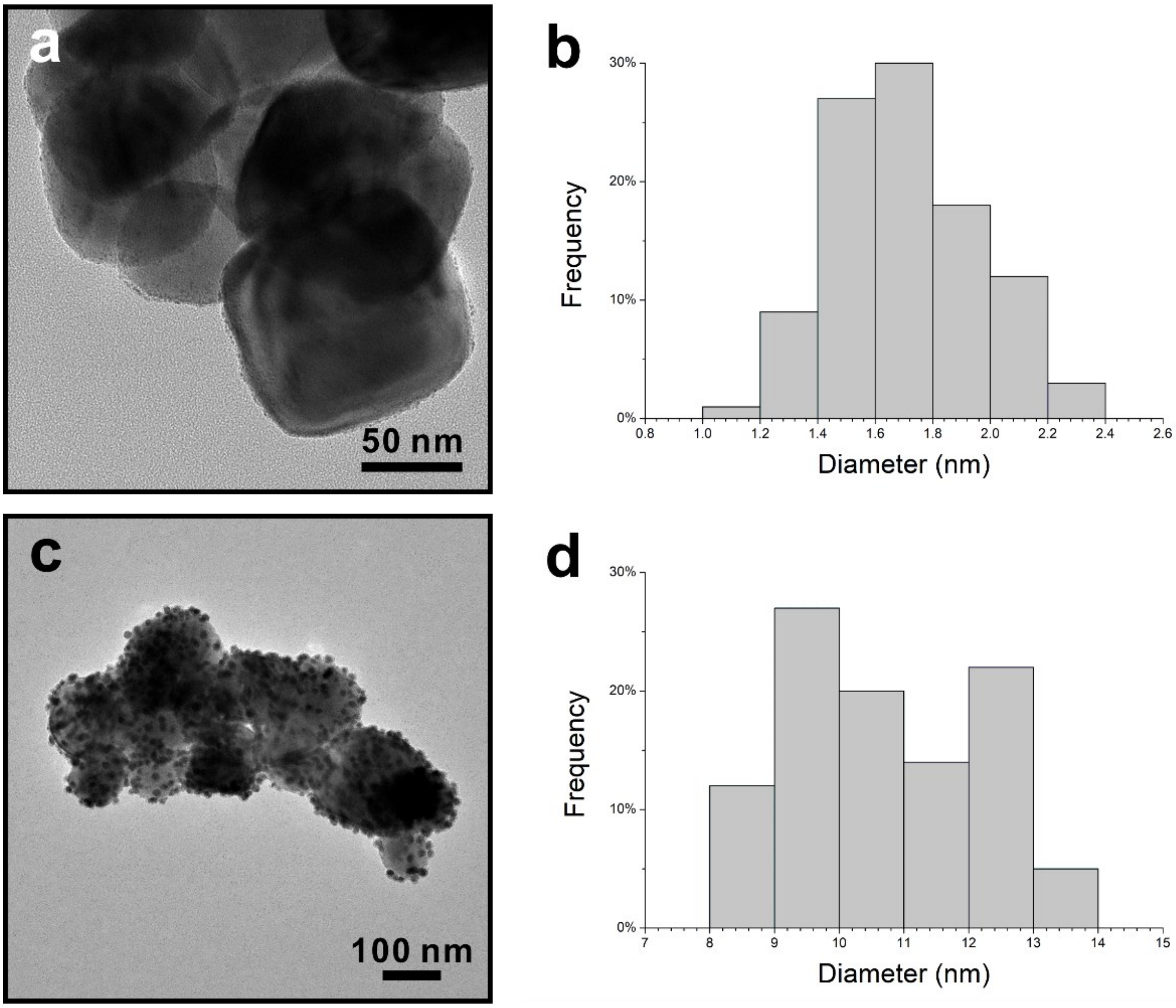

Figure S7. a) TEM image and b) size distribution of Au-BTO hybrid NPs after 30 minutes of reaction. c) TEM image and d) size distribution of Au-BTO hybrid NPs after 4 hours of reaction.

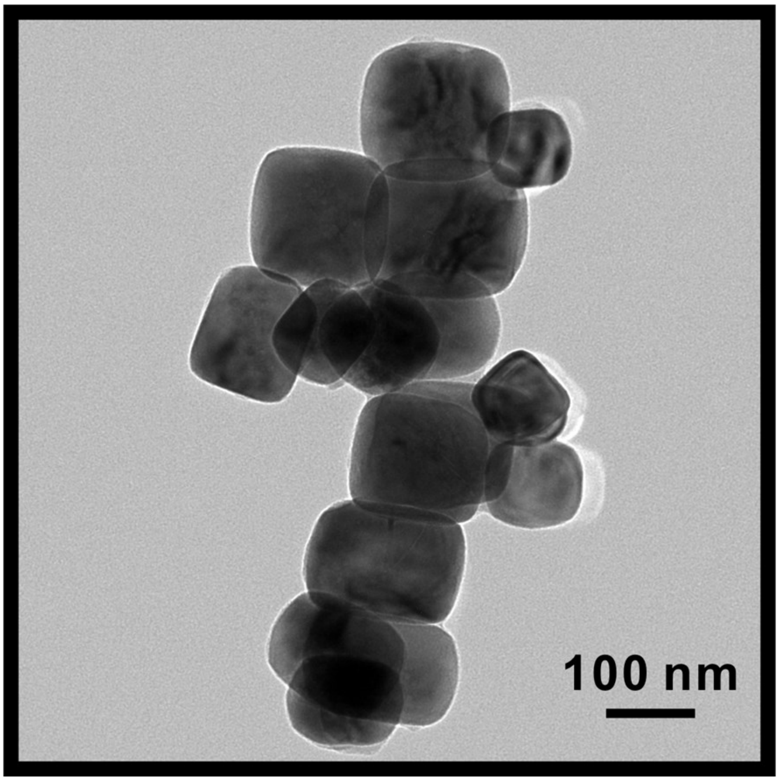

Figure S8. TEM image shows that Au/Pt bimetallic nanoparticles cannot be synthesized using $HAuCl_4$ and K_2PtCl_4 as precursors after 2-hour reaction without temperature oscillation.
